# The first Brazilian bovine breed: structure and genetic diversity of the Curraleiro Pé-duro

**DOI:** 10.7717/peerj.14768

**Published:** 2023-04-11

**Authors:** Mérik Rocha-Silva, José Lindenberg Rocha Sarmento, Fábio Barros Britto, George Vieira do Nascimento, Lilian Silva, Geandro Carvalho, Geovergue Rodrigues de Medeiros

**Affiliations:** 1 Animal Science, Universidade Estadual do Piauí, Teresina, Piauí, Brasil; 2 Animal Science, UFPI—Universidade Federal do Piauí, Teresina, Piauí, Brasil; 3 Biology Department, UFPI—Universidade Federal do Piauí, Teresina, Piauí, Brasil; 4 Animal Production Department, INSA—Instituto Nacional do Semiarido, Campina Grande, Paraíba, Brasil

**Keywords:** AMOVA, Microsatellites, Slatikin’s genetic distance, Native breed

## Abstract

**Background:**

The production of animal-based foods from native breeds have a synergistic relationship with the regional culture, the local climate, and mainly the maintenance of alternative genetic resources for a system with a lower environmental impact. Thus the efficiency of conservation and production depends on assessing the variability of these local breeds. In the case of Curraleiro Pé-duro cattle, the most adapted individuals have undergone natural selection over five hundred years in the Brazilian savannas, mating with little or no human interference. The peculiarities of these biomes, where the regional flora is the food base and cattle is raised in extensive areas, likely influenced the genetic composition of the different groups that make up the first cattle breed of Brazil.

**Methods:**

To evaluate the composition, diversity, variation, differentiation, and genetic structure of the populations studied, samples of hair follicles from 474 individuals of different animal categories (calves, yearlings, heifers, cows, and bulls) from three farms, defined as subpopulations “A”, “B”, and “C”, were collected. The animals were genotyped for 17 microsatellite markers using a DNA sequencer. After verification of monomorphic alleles, alleles outside the expected size range, and for the presence of stutter bands, the results were subjected to statistical analysis.

**Results:**

The markers used were suitable for the proposed application with a mean Polymorphism Information Content (PIC) of 0.62. On average, the effective alleles were 4.25 per marker, with mean heterozygosities of 0.74 (observed and expected), which was lower in herd A (0.70) in comparison to herds B (0.77) and C (0.74). The analysis of molecular variance (AMOVA) revealed a higher rate of variation within herds (98.5%) and lower among herds (1.5%) (F_ST_ranging from 0.00723 and 0.03198; *p*-values < 0.05). However no significant differences among herds where found with the Mantel test based on geographic distances. The formation of genetic clusters of all animals sampled with the software Structure resulted in minimum cluster values, with two main genetic groups (*K* = 2) observed among the evaluated animals. Therefore, based on PIC and heterozygosity values, a wide genetic diversity was observed, despite little differences in population structure (AMOVA, F_ST_, and Structure results) among sampling sites.

## Introduction

Food security once again has become a global priority, as the world population is estimated to peak in the next 40 years, requiring food to be produced in quantity and quality accessible to 9.7 billion people, while preserving genetic resources (Vollset et al., 2020). Among all breeds used in animal agriculture, 17% are at risk of extinction, while 58% lack information regarding size and genetic structure (Cao et al., 2021), creating even more challenging conditions for maintaining species diversity and genetic groups in the face of human demands.

The clustering of similar individuals and the consequent emergence of genetic groups of domestic animals are associated with human and environmental dynamics. The Curraleiro Pé-duro (CPD) is descendant of the first cattle raised in the Americas and part of the history of colonization of the cerrado and caatinga (Carvalho et al., 2001). These biomes have the most challenging conditions for animal production in Brazil, with the lowest precipitation levels and highest annual temperatures.

Hartl & Clark (2010) discussed the random crossing of some characteristics, which may have occurred in CPD. This suggests that similar animals have a higher rate of inbreeding, resulting in increased homozygosity that may lead to the formation of subpopulations.

The types of crossing and environmental variables may affect the population structure or population subdivision of these naturally occurring groups, with genetic differentiation due to variations in allele frequencies among different subpopulations. Many factors can influence the formation of subpopulations including geographic location and parental origin (Carvalho et al., 2022).

The conservation and use of genetic resources require genetic diversity. The Curraleiro Pé-duro (CPD) cattle are genetically distinct from other breeds, with substantial differences mainly due to its interaction with the environment (Carvalho et al., 2022).

Also, nearly 500 years of uncontrolled mating with the formation and extinction of subpopulations had repercussions on its current genetic composition (Carvalho et al., 2022; Oliveira, 2008).

The genetic diversities of animal resources can enhance or limit their conservation and/or use for food production in the face of global challenges. This study was aimed at evaluating the composition, diversity, variation, differentiation and genetic structure of the Curraleiro Pé-duro cattle raised in the states of Maranhão and Piauí, where it originated and evolved.

## Materials & Methods

### Sampling and collection of biological material

The sampling of the specimens used in this study was designed to avoid the Wahlund effect (Meeûs, 2018), and consisted of 474 individuals, representing approximately 10% of the known population of Curraleiro Pé-duro cattle.

Hair follicle samples from males and females were taken from various categories (calves, yearlings, cows and bulls), with the adults registered as base herd with the Associação Brasileiro de Criadores de Curraleiro Pé-duro.

The database is available at https://doi.org/10.5281/zenodo.7115391

The three herds visited (herein referred to as farms or subpopulations “A”, “B”, and “C”) are participants in the genetic improvement program of the breed, conducted by ABCPD, the Federal University of Piauí (FUP), and the State Univerity of Piauí (UESPI). This research project has been approved by the Research Ethics Committee of the Federal University of Piauí under #683/21 CEUA-UFPI. Farm “A” is located in the Carnaubais region of Piauí (−4.652482218991185, −42.05797250656697), Farm “B” in the Cocais region of Maranhão (−5.231024540320055, −44.47868128067367) and Farm “C” in the Sambito Valley of Piauí (−6.082350629527833, −42.24258929956412). Each farm represents a subpopulation, and exchanges of a small number of individuals occur irregularly and sporadically among farms, during the process of commercialization of animals.

### Genotyping with microsatellite markers

PCR products were obtained with primers marked with fluorophores and, after purification, were examined with a Thermo Fisher ABI 3730 DNA analyzer (Thermo Fisher, Waltham, MA, USA). Fragment size was determined with the GeneMapper^®^ program, generating a file with the alleles present in each animal, for each marker tested.

The genotyping of the animals was outsourced to Laboratório Raça (Goiânia, Brazil), accredited by the Ministry of Agriculture, Livestock and Supply, Brazil (MAPA).

Seventeen microsatellite markers were used (Table 1) following MAPA #45 of 12/15/2017 (MAPA BRASIL, 2017), and in agreement with the results of comparative tests carried out by the International Society of Animal Genetics (ISAG). These markers are suitable for the investigation of the genetic structure, and the optimization of the findings aimed at the applicability of individual results in the identification of kinship, useful in the structuring of the breed that currently has only a base herd.

**Table 1:** Microsatellites used.

**Microsatellite**	**Chromosomal location**	**Sequency**	Primer forward Primer reverse	Reference	Accuracy % (ISAG, 2021)
BM1818	D23S21	(TG)n	AGCTGGGAATATAACCAAAGG AGTGCTTTCAAGGTCCATGC	1	99.78
BM1824	D1S34	(GT)n	GAGCAAGGTGTTTTTCCAATC CATTCTCCAACTGCTTCCTTG	2	98.64
BM2113	D2S26	(CA)n	GCTGCCTTCTACCAAATACCC CTTCCTGAGAGAAGCAACACC	3	98.26
*CSRM60*	D10S5	(AC) _n_	AAGATGTGATCCAAGAGAGAGGC AAGGACCAGATCGTGAAAGGCATAG	4	^*^
CSSM66	D14S31	(AC)n	ACACAAATCCTTTCTGCCAGCTGA AATTTAATGCACTGAGGAGCTTGG	2	^*^
ETH3	D19S2	(GT) _n_AC(GT)_6_	GAACCTGCCTCTCCTGCATTGG ACTCTGCCTGTGGCCAAGTAGG	5	98.75
ETH10	D5S3	(AC)n	GTTCAGGACTGGCCCTGCTAACA CCTCCAGCCCACTTTCTCTTCTC	5	98.26
ETH225	D9S2	(TG)4CG(TG)(CA)n	GATCACCTTGCCACTATTTCCT ACATGACAGCCAGCTGCTACT	6	96.73
ILSTS006	D7S8	(GT)n	TGTCTGTATTTCTGCTGTGG ACACGGAAGCGATCTAAACG	7	^*^
INRA23	D3S10	(AC)n	GAGTAGAGCTACAAGATAAACTTC TAACTACAGGGTGTTAGATGAACTC	8	98.75
SPS113	BTA10		CCTCCACACAGGCTTCTCTGACTT CCTAACTTGCTTGAGTTATTGCCC	–	^*^
SPS115	D15	(CA)nTA(CA)6	AAAGTGACACAACAGCTTCTCCAG AACGAGTGTCCTAGTTTGGCTGTG	4	99.46
TGLA53	D16S3	(TG)6CG(TG)4(TA)n	GCTTTCAGAAATAGTTTGCATTCA ATCTTCACATGATATTACAGCAGA	9	98.58
TGLA57	BTA1	(GT)n	CTAATTTAGAATGAGAGAGGCTTCT TTGGTCTCTATTCTCTGAATATTCC	9	^*^
TGLA122	D21S6	(AC)n(AT)n	AATCACATGGCAAATAAGTACATAC CCCTCCTCCAGGTAAATCAGC	9	98.09
TGLA126	D20S1	(TG)n	CTAATTTAGAATGAGAGAGGCTTCT TTGGTCTCTATTCTCTGAATATTCC	9	97.54
TGLA227	D18S1	(TG)n	CGAATTCCAAATCTGTTAATTTGCT ACAGACAGAAACTCAATGAAAGCA	9	97.30

**Notes.**

* Not tested by ISAG for cattle. 1 Bishop et al. (1994); 2 Barendse et al. (1994); 3 Sunden et al. (1993); 4 Baylor College of Medicine Human Genome Sequencing Center (2006); 5 Solinas-Toldo et al. (1993); 6 Steffen et al. (1993); 7 Brezinsky, Kemp & Teale (1993); 8 Vaiman et al. (1994); 9 Georges & Massey (1992).

### Data analysis

Micro-Checker 2.2.3 software (Van Oosterhout et al., 2004) was used to check for errors in the genotyping and tabulation of results, including the verification of possible monomorphic alleles, alleles outside the expected size range and the presence of stutter bands. Following this quality control, the data were analyzed based on the calculation of PIC, Hardy-Weinberg equilibrium test with Bonferroni correction at 5% significance level; and the estimation of null allele frequency performed with Cervus 3.0.7 software (Kalinowski, Taper & Marshall, 2007).

The number of alleles (Na), effective alleles (Ne), and expected (He) and observed (Ho) heterozygosity were estimated with the GenAIEx 6.5 software (Peakall & Smouse, 2012).

Wright’s F statistics (Wright, 1949) were carried out using the Gene-Pop Package version 4.7.5 (Rousset, 2022) of R 4.2 (R Core Team, 2022).

To examine differences among individuals and populations, the analysis of molecular variance (AMOVA) by Excoffier, Smouse & Quattro (1992) was performed based on the allele frequency of haplotypes with a nonparametric statistical test of permutations (Excoffier, Smouse & Quattro, 1992), using the Arlequin 3.5.2.2 software (Excoffier & Lischer, 2010) configured to carry out 10 thousand permutations using ΦST values.

Genetic differences among populations were examined as a function of the geographic distances among them using the Mantel test. Geographic distances among populations (Table 2) were log transformed to linearize the matrix of genetic distance and distance among populations (Smith & Weissman, 2020). In this test, the Slatikin’s genetic distance used was based on 10 thousand permutations in Arlequin 3.5.2.2 software (Excoffier & Lischer, 2010).

**Table 2:** Geographic locations and road distances in km on the lower diagonal and logarithmized on the upper diagonal.

Subpopulations	Latitude Longitude	A	B	C
A	−4652482218991180 −4205797250656690	0	2.50379068	2.38381537
B	−5231024540320050 −4447868128067360	319	0	2.54654266
C	−6082350629527830 −4224258929956410	242	352	0

Structure 2.3.4 (Pritchard, Stephens & Donnelly, 2000) was used to perform a cluster analysis based on Bayesian statistics using the Monte Carlo Markov Chains method (MCMC) according to the similarity of genotypes, evaluating number of possible genetic groups (K) ranging from 1 to 10. The program was set to run 500,000 MCMC samples with 100,000 burn-in iterations (400,000 for inference) and for each run as indicated by Gilbert et al. (2012) on 20 repetitions.

Because animals are exchanged among properties (subpopulations), the program was adjusted to run the admixture model (Admixture) that aims to infer a single Alpha and use it for all populations. However, it was tested with an alpha estimate for each population (farm) (Data S1).

The results were analyzed using the main interpretive methodology by Evanno, Regnaut & Goudet (2005), and the most recently recommended method by Puechmaille (2016).

The graphical representation of the results was carried out using the Structure Selector software (Li & Liu, 2018).

## Results

### Analysis of STR usability and data integrity

Seventeen microsatellite loci showed high level of polymorphism to evaluate the genetic diversity of the sampled populations (Table 3), with a mean PIC of 0.6184, considered as very informative (PIC >0.50) by the classification of Botstein et al. (1980).

**Table 3:** Quality indicators of microsatellite markers selected for the sampled population.

**Microsatellite**	**PIC**	**An**		**Microsatellite**	**PIC**	**An**
BM1818	0.682	0.0207		INRA23	0.649	0.0226
BM1824	0.698	−0.0169		SPS113	0.805	0.0321
BM2113	0.768	0.0216		SPS115	0.594	0.0329
CSRM60	0.677	0.0141		TGLA53	0.834	−0.0089
CSSM66	0.742	0.0251		TGLA57	0.748	0.0387
ETH3	0.789	0.0068		TGLA122	0.785	0.0002
ETH10	0.651	0.0000		TGLA126	0.552	0.0623
ETH225	0.746	0.0170		TGLA227	0.766	−0.0017
ILSTS006	0.667	0.0087		–	–	–

**Notes.**

PIC polymorphism information content An Estimated null allele frequency

Indications of null alleles were identified in only a portion of the loci used and in low incidence ($\bar {y}=0.016$). Therefore, most were real alleles, with a low incidence of null alleles (An). The highest incidence was in TGLA126, with an estimate of zero or very close to zero for others. This is an indication of the reliability of the laboratory analysis, ruling out the possibility that the genetic differentiation measures were overestimated. Evidence of null alleles was found at only a portion of the loci used, at low incidence.

### Genetic variation of subpopulations

The estimated expected heterozygosity (He) was 0.74 (Table 4).

**Table 4:** Allele numbers and heterozygosity in the three subpopulations.

**Subpopulation**	**N**	**Na**	**Ne**	**He**	**Ho**
General	474	8.41 ± 0.38	4.07 ± 0.15	0.74 ± 0.01	0.74 ± 0.01
A	237	9.47 ± 0.68	4.17 ± 0.26	0.74 ± 0.02	0.70 ± 0.02
B	64	6.71 ± 0.39	3.85 ± 0.25	0.72 ± 0.02	0.77 ± 0.03
C	173	9.06 ± 0.70	4.19 ± 0.28	0.74 ± 0.02	0.74 ± 0.02

**Notes.**

Second digit of rounded values.

Na number of different alleles Ne number of effective ales He expected heterozygosity Ho observed heterozygosity

Based on the number of heterozygous individuals, the observed heterozygosity (Ho) was estimated, and was observed to be same as He.

### Genetic differentiation among animals of the Curraleiro Pé-duro breed

The fixation indices proposed by Wright (Wright, 1949) are shown in Table 5.

**Table 5:** Fixation indices by loci in Curraleiro Pé-duro breed.

*Loci*	Range (bp)	Ho	He	*F*_*IS*_	*F*_*ST*_	*F*_*IT*_
BM1818	258–282	0.70	0.72	0.0423	0.0007	0.0430
BM1824	178–192	0.77	0.74	−0.0306	−0.0015	−0.0321
BM2113	125–145	0.76	0.79	0.0432	0.0027	0.0458
CSRM60	92–118	0.69	0.71	0.0333	0.0014	0.0346
CSSM66	135–199	0.81	0.82	0.0146	0.0012	0.0158
ETH3	103–127	0.66	0.68	0.0029	0.0086	0.0115
ETH10	209–223	0.81	0.63	−0.1604	0.2565	0.1372
ETH225	140–158	0.72	0.71	0.0200	0.0026	0.0226
ILSTS006	282–304	0.65	0.66	0.0436	0.0090	0.0522
INRA23	194–218	0.72	0.75	0.0435	0.0030	0.0464
SPS113	135–157	0.78	0.82	0.0589	0.0034	0.0621
SPS115	246–260	0.63	0.64	0.0550	0.0146	0.0687
TGLA53	152–188	0.85	0.83	−0.0181	0.0054	−0.0126
TGLA57	084–102	0.73	0.77	0.0796	−0.0030	0.0769
TGLA122	137–177	0.79	0.79	0.0011	0.0057	0.0068
TGLA126	115–125	0.53	0.61	0.1149	0.0066	0.1207
TGLA227	77–97	0.79	0.79	−0.0009	0.0054	0.0045

**Notes.**

Bp base pair Ho Observed Heterozygosity He Expected Heterozygosity *F*_IS_ Inbreeding coefficient within individuals *F*_ST_ Inbreeding coefficient within subpopulations, relative to the total *F*_IT_ Inbreeding coefficient of the total population N 474 animals

Because *F*_IS_ is the product of the difference between He and Ho in relation to He, it indicates whether there are changes in population structure. Using a population in Hardy-Weiberg equilibrium (E-HW) as a model, the expected heterozygosity of the subpopulations (Farms A, B, C) was higher than expected in a theoretical population (Table 5). The low values obtained suggest a population close to equilibrium.

### Genetic structure of the sampled population

The molecular analysis of variance (AMOVA) revealed the degree of genetic structure of the subpopulations sampled (Table 6).

**Table 6:** Analysis of Molecular Variance (AMOVA).

Sources of variation	DF	Sum of squares	Variation components	Variation percentage
Among subpopulations	2	67.058	0.09593^***^	1.50
Intra subpopulations	945	5964.906	6.31207	98.50
Total	947	6031.964	6.40800	

**Notes.**

DF, Degrees of Freedom.

*** *p* < 0.001.

The 98.5% of the variation arising from individuals within populations suggests that there is greater variation within the herd than among herd. Thus herds are relatively similar, with greater variation occurring among the individuals that make up each herd. This condition mischaracterizes the degree of genetic structuring among sampled population.

In general, the population analysis revealed a low structure in each sampled location (Table 7), but the *F*_ST_ values found were significant (*P*-value <0.05). Despite being low, the value observed for the population of Maranhão was higher than that of the populations of Piauí compared between them (Table 7). However, genetic distance might not be associated with geographic distance because the results of the Mantel test were not significant (Table 8) (*p*-value > 0.05.

**Table 7:** Nei genetic distance (lower diagonal) and *F*_ST_ distance (upper diagonal).

Population	**A (PI)**	**B (MA)**	**C (PI)**
Subpopulations A (PI)	0	0.02303^*^	0.00723^*^
Subpopulations B (MA)	0.061	0	0.03198^*^
Subpopulations C (PI)	0.082	0.055	0

**Notes.**

* *p* < 0.05.

**Table 8:** Matrix of logarithmized geographic distances (X, lower diagonal) and Slatikin’s genetic distance (Y, upper diagonal).

Identification	**Distance to A**	**Distance to B**	**Distance to C**
Subpopulations A	0	2.50379068	2.38381537
Subpopulations B	0.02359	0	2.54654266
Subpopulations C	0.00730	0.03304	0
Determination Coefficient R2 (Y by X, determines genetic distance by geographic distance)			0.986836
*p*-value			0.1705

*F*_ST_ ranging from 0.00723 and 0.03198); *p*-values <0.05)

Regarding differences among populations, despite AMOVA showing that the difference among herds is much smaller compared to intra-herd differences, there are indications that the genetic distance between the herd of Maranhão and the herds of Piauí is greater, based on Nei’s distance, statistically significant by *F*_ST_ (Table 7).

Genetic distance is not associated with geographic distance according to the Mantel test (Table 8).

Although almost the entire genetic distance among groups is explained by geographic distance, it is not statistically significant (*p* > 0.05). It should be pointed out that there are no other variables that notoriously affect the distance among groups (subpopulations). Therefore, there is no evidence of isolation by geographic distance.

### Genetic differences among individuals

For the clustering of individuals according to similarity of genotypes, simulations of groupings were carried out with the Structure software (Pritchard, Stephens & Donnelly, 2000), testing variations in configurations, including disregarding the possibility of permutation among populations (farms) given that the commercialization of cattle among properties varies. However, convergence was observed when using independent alphas (CLUMPAK in supplements).

**Figure 1:**
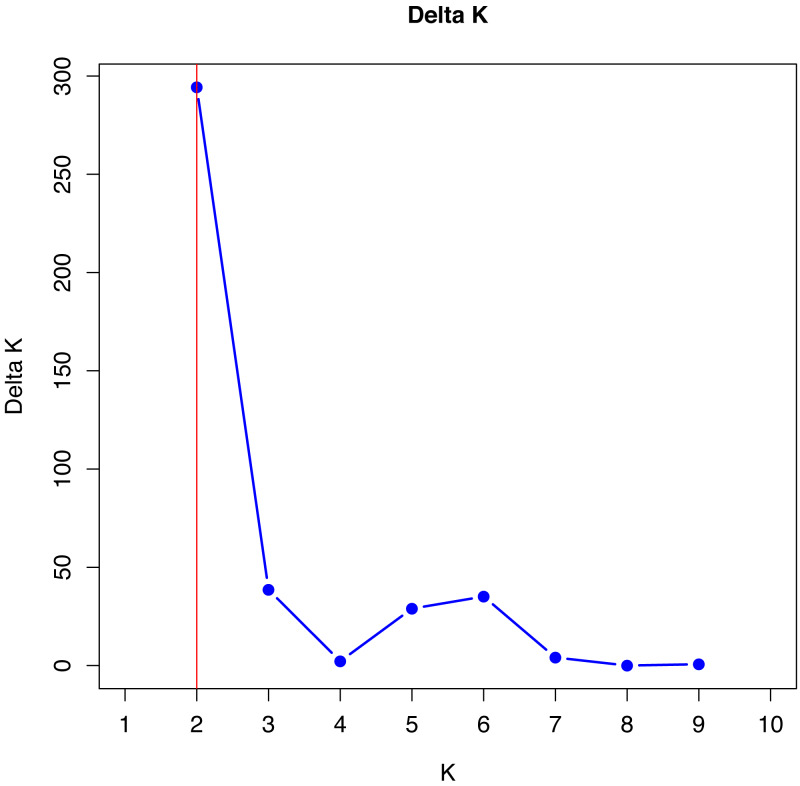
Verification of better genetic grouping of Curraleiro Pé-duro individuals as a function of Evanno’s Delta *K* values (Evanno, Regnaut & Goudet, 2005).

No single cluster was able to perfectly describe all the variability found in the sampled subpopulations. The simulation data analyzed by the method described by Evanno, Regnaut & Goudet (2005), which is based on Delta K values, indicated that the best grouping of genotypes occurs in two clusters (Fig. 1).

Gilbert et al. (2012) proposed the use of other methods of interpretation of structure simulations, especially when obtaining *K* = 2 with microsatellite data, such as expanding the methodologies for analyzing the results of the simulations, in order to avoid spurious clusters that erroneously project the value of K.

The values shown in Table 9 are in agreement with the number of clusters obtained with the method proposed by Evanno, Regnaut & Goudet (2005).

**Table 9:** Optimal K after by the method (Puechmaille, 2016).

**K**	**MedMed**	**MedMean**	**MaxMed**	**MaxMean**	**Reps**
1	1	1	1	1	20
2	2	2	2	2	20
3	1	1	1	1	20
4	1	1	1	1	20
5	1	1	1	1	20
6	1	1	1	1	20
7	1	1	1	1	20
8	1	1	1	1	20
9	1	1	1	1	20
10	0	1	1	1	20
	MedMedK	MedMeaK	MaxMedK	MaxMeaK	
	2	2	2	2	

Therefore, in both methods, *K* = 2 was the appropriate cluster number to describe the number of subpopulations. The 10 simulations converged in the 20 trials performed with *K* = 2 (see supplement) indicating the same cluster, following Kopelman et al. (2015) for clusters that showed greater convergence.

**Figure 2:**
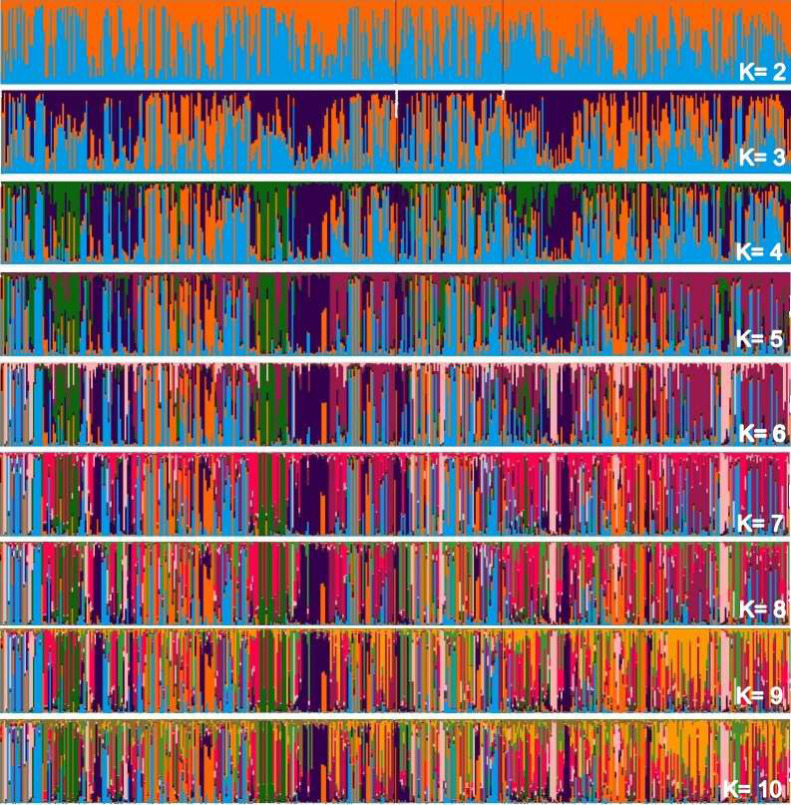
Main groups of subpopulations of Curraleiro Pé-duro cattle detected by the method of determining clusters of genetic similarity.

Considering two main groups, the participation of each individual (vertical line) within each rectangle was evaluated into subgroupings in Fig. 2, which makes up the individualized grouping for each ΔK.

The genetic compositions of each of the subpopulations within each cluster is shown in Table 10, detailing the information in the above graph.

**Table 10:** Genetic participation of populations for each group (cluster).

	**N**	**Cluster 1**	**Cluster 2**
Subpopulation A (PI)	237	0.4819	0.5181
Subpopulation B (MA)	64	0.3634	0.6366
Subpopulation C (PI)	173	0.5831	0.4169

## Discussion

Deficits of heterozygotes as a function of null alleles affect the estimation of Wright’s *F* (*F*_ST_, *F*_IS_, and *F*_IT_), compromising the estimation of the causes of deviations from the expected Hardy–Weinberg genotypic proportions, among others (Meeûs, 2018).

For animals of the same breed, Egito et al. (2007) and Silva Filho et al. (2014) reported a lower PIC, while (Oliveira, 2008) obtained a higher value (0.723) than those found in the present study. In all these studies, the numbers of individuals sampled were much smaller, and some of the microsatellite markers used were different. Thus, when choosing microsatellite markers, in addition to the strength of the link between the marker and the genomic region (Jahnke, Smidla & Poczai, 2022), the quality endorsed by organizations such as ISAG, and how polymorphic they are in each population should be considered as well.

Expected heterozygosity (He) is the most frequently used indicator for genetic diversity analysis, as it is associated with other non-estimated indicators such as the proportion of polymorphic loci because they are interrelated (Eckert, Samis & Lougheed, 2008). Since animal management does not use technical criteria for breeding, the individuals studied could have a high frequency of inbreeding, which was not observed. This expected heterozygosity (He) estimated at 0.74 (Table 4) refers to the high chance (74%) of an animal chosen at random among CPD cattle to present a heterozygous genotype for the selected markers based on the Hardy-Weinberg theorem.

Egito et al. (2007) found a reduced heterozygosity in native (or creole) breeds of Brazilian cattle as a result of the influence of other subspecies (*Bos taurus indicus*) and inbreeding within herds, an unfavorable condition for genetic diversity. In the case of CPD, heterozygosity was higher (0.74), indicating a greater level of variation.

This discussion is relevant, and expansion with new microsatellites to replace less informative markers should be carried out. Investigations like the present one applied little-used markers, that in the case of CPD were very informative (estimated PIC), such as five of the loci used (CSRM60, CSSM66, ILSTS006, SPS113 and TGLA57).

Comparing with the results of *F*_IS_ per loci reported by Silva Filho et al. (2014) and Oliveira et al. (2012), of the 11 microsatellites used in common, Silva Filho et al. (2014) obtained five with lower inbreeding coefficients. The animals kept by EMBRAPA in São João do Piauí were one of the main sources for the formation of the sampled herds, therefore given the genealogical contribution of the animals sampled by Silva Filho et al. (2014) for the formation of herds in subpopulations (farms) A, B and C, similarities are expected.

Silva Filho et al. (2014) and Oliveira et al. (2012) reported a loss of genetic variability in the Curraleiro Pé-duro breed due to inbreeding in the last decade, mainly because herds are kept only for conservation purposes, and suggested increasing the number of animals. Considering the discussed by Silva Filho et al. (2014) and the results obtained in the present study revealed a comparatively better scenario, as the increase in the number of animals reduced homozygosity, improving variability. Therefore, the development of commercial herds has promoted the conservation of the breed.

Artificial selection (not necessarily based on technical criteria) tends to interfere with genetic diversity, which was not observed. Reduced inbreeding rates was found in the sampled population, similar to that identified by Rovelli et al. (2021), when the high use of bulls contributes to lower kinship coefficients. These authors pointed out the importance of minimizing inbreeding, especially in the current scenario of intense and rapid increase in inbreeding in cattle populations under intense selection associated with reproductive biotechnologies. In the case of Italian cattle, production was improved while maintaining control of inbreeding levels.

The locus ETH10 had the highest estimated *F*_ST_, with the mean among the 17 markers being 0.015 (± 0.010), which according to the classification by Hartl & Clark (2010), indicates that the differentiation among loci in the sampled subpopulations is small.

Other researchers working with the same breed (CPD) identified the presence of three genetic groups Freitas et al. (2021), with most individuals from Piauí forming a group in a different cluster than animals from Tocantins. In our investigation, animals from another region not sampled in previous studies, Maranhão, were included. However, there is no evidence of subpopulations among the sampled animals, which were grouped in a similar way to the animals raised in the Carnaubais region of Piauí (Campo Maior and Cocal de Telhas) and Vale do Sambito (Elesbão Veloso).

The number of K can be differ depending on the methodology and the most common one is based on ΔK, used in all studies cited (Silva Filho et al., 2014; Freitas et al., 2022) and that indicated two genetic groups among Curraleiro Pé-duro cattle currently raised in the mid-northern region of Brazil (*k* = 2). However, Gilbert et al. (2012) suggested more suitable methods such as the proposed by Puechmaille (2016) that used the estimators: medmedk, medmeak, maxmedk and maxmeak, as they are considered more accurate than the LnPr(X — K) and ΔK method. Both methodologies were applied and they corroborated the same results, supporting the existence of two main groups (*k* = 2).

Clustered alleles in the two subpopulations comprise the genotype of individuals from the three different populations (farms) sampled. There are sets of lines representing individuals with a single predominant color, for example, at two occasions in population A and another in population C with individuals grouped in purple (RGB:51,0, 75). At these points you have close individuals. This pattern can initially be visualized when Δk =3 and is maintained throughout the simulations of relatively individualized clusters from the others, suggesting secondary additional clusters in the sampled populations.

These clusters are notorious in studies with individuals of different breeds, in which clusters almost always make up a different race from the others. In the case of animals of a single breed, clusters indicate conditions for grouping individuals within the same breed into different groups based on similarities.

It should also be noted that the clusters were not predominant in any of the subpopulations, with specimens of all clusters occurring in all sampled farms.

## Conclusions

The microsatellite markers used by MAPA (Ministério da Agricultura e Pecuária - Brazil) to assess kinship in bovine species were highly polymorphic and suitable for studies of structure and genetic diversity.

Some little known markers proved to be highly efficient in the variability of genotypes among the population studied.

Heterozygosities were high for all loci in the three subpopulations, including excess heterozygosity in some cases, which resulted in low inbreeding rates (*F*_IS_).

Most of the variability is within rather than among herds. However, genetic distance was greater between the Maranhão and the Piauí herds than between the Piauí herds sampled based on ΦST.

A greater portion of the genetic variation was observed among individuals, with no relevant variation among populations, genotypic or geographic, maintaining at least two clusters, sufficient to properly group most of the genotype diversity, regardless of population.

No significant differences were found among the three populations, as animals from Maranhão had a similar genetic composition to those from Piauí, and the genetic variability was mainly associated with individuals rather than populations.

##  Supplemental Information

10.7717/peerj.14768/supp-1 Supplemental Information 1 Raw data from microsatellite genotyping The number of base pairs organized by microsatellites (columns) for the genotyped animals (rows). Format compatible with some software, such as Structure (Pritchard, 2002)

10.7717/peerj.14768/supp-2 Supplemental Information 2 Coat diversity and details of the variety of hair pigmentation identified in Curraleiro Pé-duro cattle

10.7717/peerj.14768/supp-3 Supplemental Information 3 Parameters assumed in the Software Structure in five rounds verifying that it is more appropriate to use a single alpha for the three subpopulations sampled and that the number of Burnin and Interactions assumed in the fourth round of analysis is adequate

## Additional Information and Declarations

### Data Availability

The following information was supplied regarding data availability: The data is available at Zenodo: Mérik Rocha Silva. (2022). Microssatelite CPD [Data set]. Zenodo. https://doi.org/10.5281/zenodo.7115391.
