# Supplementary material for: The first Brazilian bovine breed: structure and genetic diversity of the Curraleiro Pé-duro
Source: PeerJ. 2023 Apr 11;11:e14768. doi: 10.7717/peerj.14768 (PMC10103694; doi:10.7717/peerj.14768)

Frontal images of some animals sampled to illustrate the appearance of Curraleiro Pé-Duro cattle. (Photographer Mérik Rocha Silva)

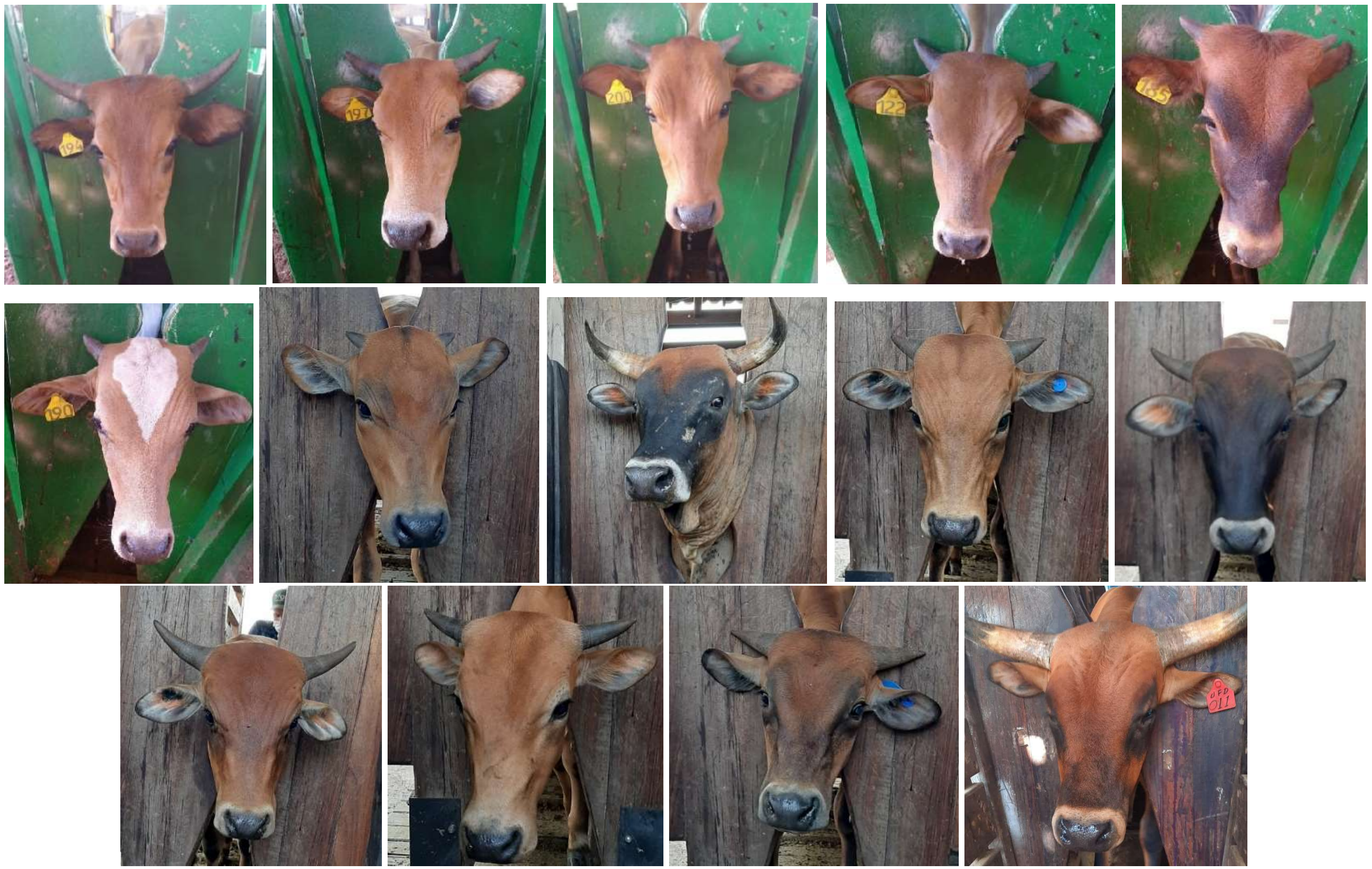

Supplement: Supplemental Information 2 [file peerj-11-14768-s002.pdf]
