# Supplementary material for: The first Brazilian bovine breed: structure and genetic diversity of the Curraleiro Pé-duro
Source: PeerJ. 2023 Apr 11;11:e14768. doi: 10.7717/peerj.14768 (PMC10103694; doi:10.7717/peerj.14768)
Supplement: Supplemental Information 3 [file peerj-11-14768-s003.pdf]

Parameters tested in Software Structure and CLUMPAK main pipeline summary

Parameter	Analysis round				
	**1^{ft}**	**2nd**	**3rd**	**4th**	**5th**
Burnin	1,000	10,000	10,000	100,000	200,000
interactions (MCMC)	5,000	50,000	50,000	500,000	1,000,000
Single alpha (*Admixture Model*)	Yes	Yes	No	Yes	Yes
Interactions	20	20	20	20	20
K=1	20/20	20/20	20/20	20/20	20/20
K=2	20/20	20/20	20/20	20/20	20/20
K=3	10/20, 10/20	11/20, 9/20	11/20, 9/20	11/20, 9/20	9/20, 6/20, 5/20
K=4	12/20, 5/20, 3/20	12/20, 8/20	14/20, 5/20	**20/20**	**20/20**
K=5	9/20, 8/20, 3/20	10/20, 10/20	9/20, 6/20, 5/20	15/20, 3/20, 2/20	11/20, 9/20
K=6	17/20, 2/20	16/20, 2/20	12/20, 6/20, 2/20	**20/20**	**20/20**
K=7	18/20	**20/20**	9/20, 8/20, 3/20	20/20	20/20
K=8	14/20, 6/20	8/20, 6/20, 6/20	18/20	14/20, 4/20 2/20	14/20, 4/20, 2/20
K=9	10/20, 8/20, 2/20	11/20, 6/20, 3/20	11/20, 5/20, 2/20, 2/20,	15/20, 4/20	14/20, 2/20, 2/20
K=10	20/20	16/20, 2/20, 2/20	9/20, 6/20, 4/20	16/20, 2/20, 2/20	12/20, 4/20, 3/20
Best K (Puechmaille Method)	2	2	2	2	2
Best K (Evanno method)	7	2	2	2	2
